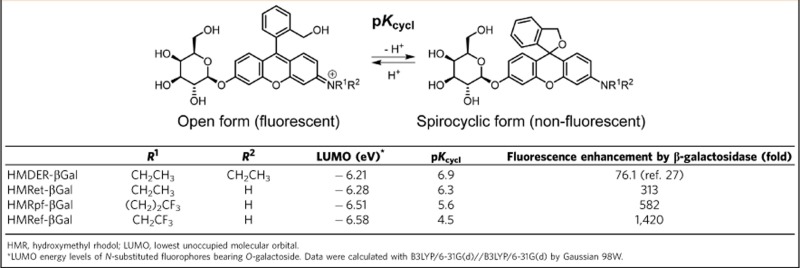# Sensitive β-galactosidase-targeting fluorescence probe for visualizing small peritoneal metastatic tumours *in vivo*

**DOI:** 10.1038/ncomms7463

**Published:** 2015-03-13

**Authors:** Daisuke Asanuma, Masayo Sakabe, Mako Kamiya, Kyoko Yamamoto, Jun Hiratake, Mikako Ogawa, Nobuyuki Kosaka, Peter L. Choyke, Tetsuo Nagano, Hisataka Kobayashi, Yasuteru Urano

**Affiliations:** 1 Graduate School of Medicine, The University of Tokyo, 7-3-1 Hongo, Bunkyo-ku, Tokyo 113-0033, Japan; 2 Graduate School of Pharmaceutical Sciences, The University of Tokyo, 7-3-1 Hongo, Bunkyo-ku, Tokyo 113-0033, Japan; 3 PRESTO, Japan Science and Technology Agency, 4-1-8 Honcho, Kawaguchi, Saitama 332-0012, Japan; 4 Institute for Chemical Research, Kyoto University, Gokasho, Uji, Kyoto 611-0011, Japan; 5 Molecular Imaging Program, Center for Cancer Research, National Cancer Institute, US National Institutes of Health, Building 10, Room B3B69, Mail Stop Code 1088, 10 Center Drive, Bethesda, Maryland 20892-1088, USA; 6 Open Innovation Center for Drug Discovery, The University of Tokyo, 7-3-1 Hongo, Bunkyo-ku, Tokyo 113-0033, Japan; 7 CREST, Japan Science and Technology Agency, 4-1-8 Honcho, Kawaguchi, Saitama 332-0012, Japan; 8 Basic Research Program, Japan Science and Technology Agency, 4-1-8 Honcho, Kawaguchi, Saitama 332-0012, Japan

## Abstract

Fluorescence-guided diagnostics is one of the most promising approaches for facile detection of cancer *in situ*. Here we focus on β-galactosidase, which is overexpressed in primary ovarian cancers, as a molecular target for visualizing peritoneal metastases from ovarian cancers. As existing fluorescence probes are unsuitable, we have designed membrane-permeable HMRef-βGal, in which the optimized intramolecular spirocyclic function affords >1,400-fold fluorescence enhancement on activation. We confirm that HMRef-βGal sensitively detects intracellular β-galactosidase activity in several ovarian cancer lines. *In vivo*, this probe visualizes metastases as small as <1 mm in diameter in seven mouse models of disseminated human peritoneal ovarian cancer (SHIN3, SKOV3, OVK18, OVCAR3, OVCAR4, OVCAR5 and OVCAR8). Because of its high brightness, real-time detection of metastases with the naked eye is possible. Endoscopic fluorescence detection of metastases is also demonstrated. The results clearly indicate preclinical potential value of the probe for fluorescence-guided diagnosis of peritoneal metastases from ovarian cancers.

## Abstract (web summary)

Surgical removal of the metastases remains a life-extending approach to ovarian cancer, but the nodules are difficult to detect. Here the authors show that a new cell-permeable probe for β-galactosidase can sensitively and specifically detect peritoneal metastases in mouse models of ovarian cancer.

## Graphical abstract

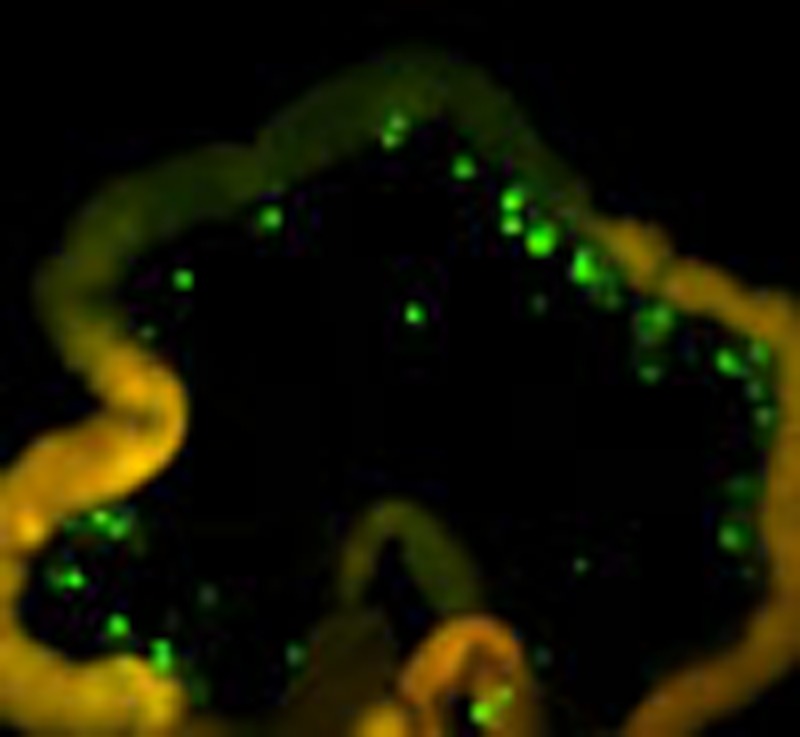

Peritoneal metastasis^1^,^2^ is associated with a wide range of malignancies, including ovarian, gastric and colorectal cancers. In particular, 60%–75% of ovarian cancer patients are diagnosed with aggressive peritoneal metastases, mainly because early diagnosis is difficult due to the asymptomatic nature of the disease in the early stages^3^,^4^,^5^. This is one reason why the prognosis is poor, with an expected 5-year survival rate of 10%–20% (ref. 4). Standard therapy consists of primary surgical cytoreduction followed by systemic chemotherapy with paclitaxel and platinum-based agents^5^. Follow-up studies have revealed that removal of peritoneal metastases as small as <1 mm in diameter improved the 5-year survival of the patients^6^,^7^. However, efficient cytoreduction is extremely difficult to achieve, owing to the poor visual contrast between cancer and normal tissues under white light.

Consequently, fluorescence-guided detection is considered one of the most promising approaches to improve cytoreduction efficacy. 5-Aminolevulinic acid has been used in clinical studies for diagnosis of malignancies including ovarian carcinoma metastases^8^, as well as malignant gliomas^9^ and bladder carcinoma^10^, but false positive rates were high^11^,^12^,^13^. As for ovarian cancers, van Dam *et al*.^14^ reported intraoperative tumour-specific imaging with a folate receptor-α-targeted probe, demonstrating clinical potential for improved staging and cytoreductive surgery. Recently, we also reported detection of metastases in preclinical mouse models using a γ-glutamyltranspeptidase (GGT) activity probe, gGlu-HMRG^15^. However, in two of the six mouse models tested, gGlu-HMRG failed to visualize metastases because of their low GGT activity^15^. This result prompted us to search for another reliable diagnostic target.

Chatterjee *et al*.^16^ reported enhanced enzymatic activities of β-galactosidase in primary ovarian cancers compared with normal ovaries. Hence, in this study we focus on β-galactosidase as an enzymatic target for fluorescence probes, to visualize metastases originated from ovarian cancers. However, existing β-galactosidase-targeting fluorescence probes are not adequate for this purpose, owing to properties such as membrane impermeability and low sensitivity. Therefore, we selected an optimized spirocyclization strategy to design a probe, HMRef-βGal, which enables highly sensitive detection of β-galactosidase activity inside living cells. We validate the potential of the synthesized probe for preclinical application and demonstrate its suitability for *in vivo* laparotomic and endoscopic detection of small peritoneal metastases in mouse models of ovarian cancer.

## Results

### β-Galactosidase activities in cancer cells

**Figure 1:**
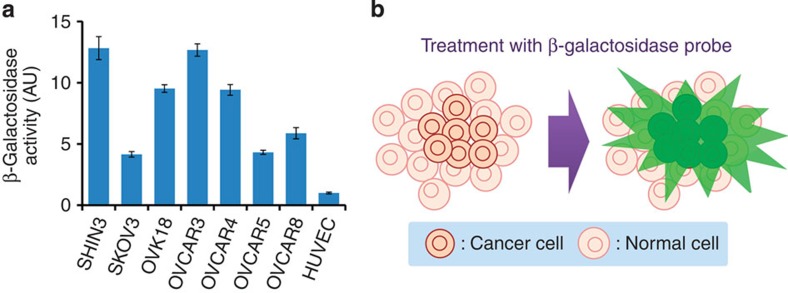
β-Galactosidase-targeting cancer visualization strategy. (**a**) β-Galactosidase activity per protein abundance in lysate of SHIN3, SKOV3, OVK18, OVCAR3, OVCAR4, OVCAR5, OVCAR8 or HUVEC cells. Data represent mean±s.d. from a single experiment in triplicate. (**b**) Schematic illustration of fluorescence detection of cancer cells with enhanced β-galactosidase activity using a fluorescence probe.

First, to confirm overexpression of β-galactosidase, we examined endogenous β-galactosidase activity in several metastatic ovarian cancer cell lines established from human ovarian cancer patients (SHIN3 (ref. 17), SKOV3 (ref. 18), OVCAR3 (ref. 19), OVCAR4, OVCAR5, OVCAR8 and OVK18 (ref. 20)). High levels of β-galactosidase activity were detected in these cell lines compared with that in a non-transformed human cell line, HUVEC (human umbilical vein endothelial cells; Fig. 1a). This result suggested that β-galactosidase is a promising target for cancer imaging (Fig. 1b).

### Molecular design and development of fluorescence probes

Therefore, we next required a suitable probe molecule. To date, various types of β-galactosidase fluorescence probes including MUG^21^, FDG^22^, RG^23^ and DAOG^24^ have been reported. However, these first-generation probes are unsuitable for live-cell imaging of intracellular β-galactosidase activity because of their membrane impermeability. Thus, we tried our second-generation membrane-permeable probe, TG-βGal^25^. However, TG-βGal could not detect intracellular β-galactosidase activity, because the fluorescent product TG was exported from the cells by organic anion transporters (see Supplementary Fig. 1 for details, including the chemical structures), which are often overexpressed in metastatic cancers and cause multidrug resistance^26^. Therefore, we next tried our recently reported HMDER-βGal^27^, whose fluorescent product HMDER has a net charge of zero. HMDER-βGal successfully detected β-galactosidase activity in cultured cancer cells (see Supplementary Fig. 2 for details, including the chemical structures), but we found that peritoneal metastases could not be specifically visualized, owing to high background fluorescence in a mouse tumour model.

We considered that the high background was probably due to the p*K*_cycl_ value of 6.9 for the spirocyclization equilibrium of HMDER-βGal^27^, because at pH 7.4 about 25% of the probe would be present in the fluorescent open form (*cf*. Table 1). Thus, to reduce the background we set out to chemically modulate the spirocyclic equilibrium to shift the p*K*_cycl_ value to ~5.4. This would imply that >99% of the probe would exist in the non-fluorescent spirocyclic form at pH 7.4. Density functional theory calculations indicated that electron-withdrawing *N*-substituents decrease the lowest unoccupied molecular orbital energy level of the fluorophore (Table 1), suggesting that the spirocyclization is stabilized.

**Figure 2:**
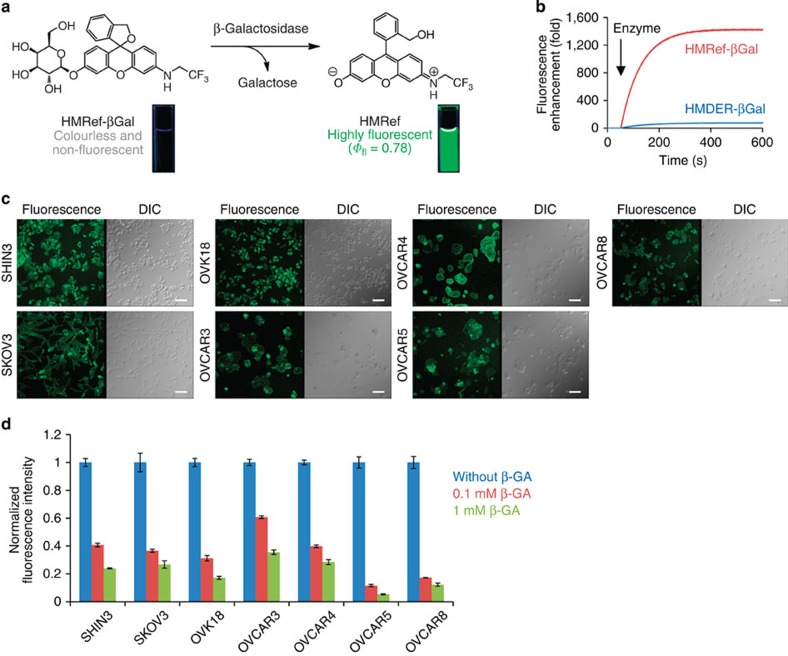
Detection of β-galactosidase activities in living ovarian cancer cells with HMRef-βGal. (**a**) Activation of HMRef-βGal on enzymatic reaction with β-galactosidase. Photographs show cuvettes containing the reaction mixture irradiated with 365-nm ultraviolet light. (**b**) Time course of enzymatic reaction of HMRef-βGal with β-galactosidase. β-Galactosidase (5 units) was added at 50 s. HMDER-βGal was used as a control. Probe concentrations were 0.5 μM. (**c**) Confocal images of ovarian tumour cells treated with HMRef-βGal. Cells were incubated with 10 μM HMRef-βGal for 1 h, and DIC and fluorescence images were obtained. Ex/Em=498 nm/505–600 nm. Scale bar, 100 μm. (**d**) Fluorescence intensity of ovarian cancer cells treated with HMRef-βGal in the presence or absence of β-GA. Fluorescence intensity of cells on a 96-well plate was measured with a plate reader (Ex/Em=498 nm/518 nm). Probe concentration was 10 μM. Data represent means±s.e.m (*n*=4).

To examine this approach, we synthesized hydroxymethyl rhodol (HMR) derivatives bearing β-galactoside and evaluated their photochemical properties (see Supplementary Tables 1 and 2, and Supplementary Fig. 3 for details). The p*K*_cycl_ values of HMRet-βGal, HMRpf-βGal and HMRef-βGal were lowered in the order of the decrease in lowest unoccupied molecular orbital energy level by the *N*-substituents, compared with HMDER-βGal (Table 1). Among them, HMRef-βGal (p*K*_cycl_=4.5) exhibited a minimal background signal at pH 7.4 and therefore showed an extremely high fluorescence enhancement by β-galactosidase (>1,400-fold) compared with HMDER-βGal (76-fold; Fig. 2a,b and Table 1). In addition, the fluorescent product HMRef (*Φ*_fl_=0.78) was much brighter than HMDER (*Φ*_fl_=0.14) (ref. 27), which would contribute to achieving high detection sensitivity of cancers. HMRef-βGal was also expected to be cell-membrane permeable, as the calculated logP of 2.61 obtained with the ALOGPS 2.1 programme^28^ was similar to that of HMDER-βGal (2.88) (ref. 27).

### Live-cell imaging of β-galactosidase

To test the performance of HMRef-βGal, we applied it to several cultured ovarian cancer cells (SHIN3, SKOV3, OVK18, OVCAR3, OVCAR4, OVCAR5 and OVCAR8). Intracellular fluorescence was detected in each case (Fig. 2c) and the fluorescence was decreased by a competitive β-galactosidase inhibitor (β-galactosylamidine; β-GA)^29^ (Fig. 2d). This result indicated that HMRef-βGal can detect intracellular β-galactosidase activity. In addition, this probe showed almost no cytotoxicity up to 100 μM in MTT (3-(4,5-dimethylthiazol-2-yl)-2,5-diphenyltetrazolium bromide) assay (Supplementary Fig. 4). We also preliminarily evaluated its toxicity to mice. A ten times higher dose than that employed for imaging application did not cause lethality or significant body weight change compared with the control group (Supplementary Fig. 5). These results suggest that HMRef-βGal may be safe for biological application, although further testing would be necessary to confirm this before the probe could be used for clinical diagnosis.

### Cancer diagnostic applications

**Figure 3:**
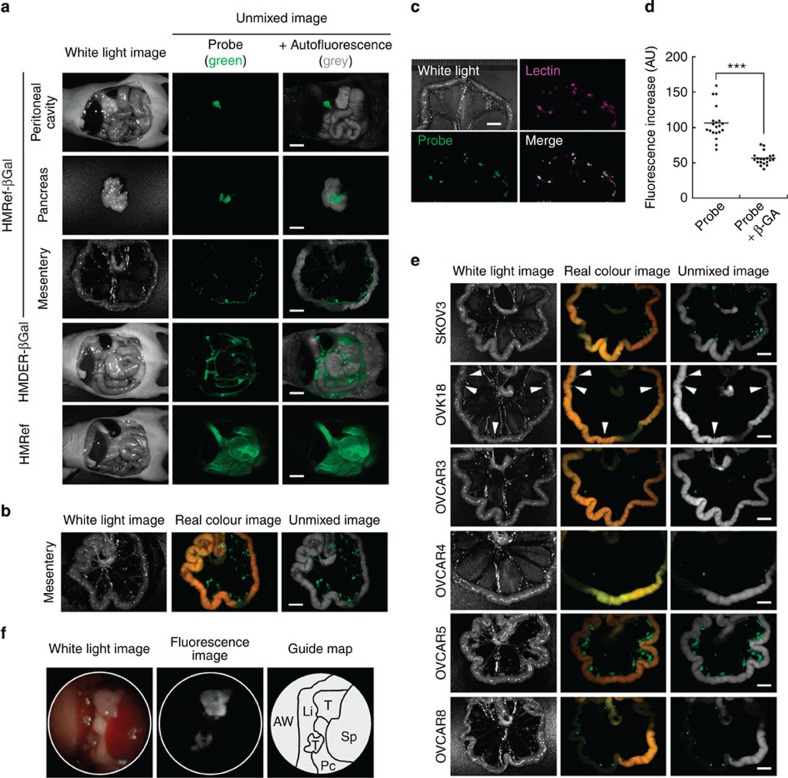
Visualization of peritoneal metastases in mouse models with HMRef-βGal. (**a**,**b**) Fluorescence spectral imaging of peritoneal SHIN3 metastasis with various probes. At 5 min (**a**) or 1 h post administration of probe (**b**), mice were immediately killed and imaged. In spectral unmixed images, fluorescence from the probe and autofluorescence were assigned as green and grey, respectively. Scale bar, 5 mm. (**c**) Dual-colour fluorescence imaging of metastases. Lectin-targeted staining was performed as a marker for metastases (Lectin). The mouse model was also treated for 1 h with HMRef-βGal and imaged (Probe). The merged image was prepared by overlaying the two unmixed images. Scale bar, 5 mm. (**d**) Fluorescence intensity on tumour nodules. In live mouse models, tumour nodules were treated with 100 μM HMRef-βGal in the presence or absence of 10 mM β-GA (see Supplementary Fig. 6). ***P<0.001 by Welch’s *t*-test (*n*=20 for each group). (**e**) Fluorescence spectral imaging of several mouse models of peritoneal metastasis at 1 h post administration of HMRef-βGal. Arrowheads indicate metastases in the OVK18 images. Scale bar, 5 mm. (**f**) Fluorescence endoscopy of tumours inside the peritoneal cavity. AW, abdominal wall; Li, liver; Pc, pancreas; Sp, spleen; T, tumour. See Supplementary Video S1 online for real-time monitoring endoscopy.

As we had confirmed that HMRef-βGal can detect intracellular β-galactosidase activity, we next examined its suitability for cancer imaging in a mouse model of peritoneal metastasis, using SHIN3 cells. At 5 min after intraperitoneal administration of the fluorescence probe, metastases as small as <1 mm in diameter inside the peritoneal cavity were clearly and specifically visualized by HMRef-βGal, but not by HMDER-βGal or β-galactoside-free HMRef (Fig. 3a). Moreover, at 1 h post administration of HMRef-βGal, the fluorescence of these nodules was enhanced so much that the tumours could be readily distinguished with the naked eye (Fig. 3b). The detected nodules were confirmed to be ovarian metastatic cancers on the basis of colocalization with a lectin-targeted stain^30^ (Fig. 3c). We also performed vital imaging and confirmed that fluorescence on the metastases was suppressed by β-GA (Fig. 3d and see Supplementary Fig. 6 for details). To check the broad availability of this probe, mouse models prepared with other cancer cell lines (SKOV3, OVK18, OVCAR3, OVCAR4, OVCAR5 and OVCAR8) were tested. We found that disseminated cancers were similarly visualized in all the models (Fig. 3e). It is particularly noteworthy that SKOV3 and OVCAR3 metastases, which could not be visualized with our previous GGT-targeted probe, gGlu-HMRG^15^, were successfully visualized with HMRef-βGal.

Next, we performed *in vivo* fluorescence endoscopy for detection of metastases. An anaesthetized mouse model pretreated with intraperitoneal administration of HMRef-βGal was subject to fluorescence laparoscopy and the metastases were successfully visualized (Fig. 3f and Supplementary Movie 1). We also performed real-time fluorescence-guided laparotomy for tumour resection. One-millimetre-sized metastases were readily recognized and resected from the peritoneal cavity (Supplementary Movie 2 and Supplementary Fig. 7). In this trial, the operator could recognize the location of metastases in a direct three-dimensional view by visible fluorescence of the probe. Thus, our developed technique with HMRef-βGal was demonstrated to have clear potential for fluorescence guidance of tumor diagnosis and surgical cytoreduction.

## Discussion

In this study, we have developed a highly sensitive β-galactosidase probe, HMRef-βGal, by chemically optimizing the intramolecular spirocyclic function. In contrast to previously reported probes, HMRef-βGal enabled sensitive detection of intracellular β-galactosidase activity in living cells. Using HMRef-βGal, we successfully imaged small peritoneal metastases in seven different mouse models, confirming the validity of β-galactosidase as a molecular target for visualizing peritoneal metastases. Importantly, this result also demonstrated the ability of our technique to broaden the diagnostic spectrum for cancers by showing that HMRef-βGal visualized SKOV3 and OVCAR3 metastases, which could not be visualized with gGlu-HMRG^15^. We confirmed that HMRef-βGal is available for laparotomic and endoscopic detection of *in vivo* metastases. Thus, our technique appears to have preclinical potential value for fluorescence-guided diagnosis of cancers with enhanced β-galactosidase activity. The β-galactosidase-based diagnostic spectrum may include not only ovarian cancer but also breast and colon cancers^31^, and gliomas^32^.

The enzymatic activation strategy is attractive for generating highly amplified fluorescence by turnover at the lesion site. We found that metastases could be visualized in as short a period as 5 min post administration. This rapid response might allow the probe to be used not only pre-operatively but also intra-operatively when suspicious lesions are encountered during diagnosis and/or surgery. Furthermore, the bright and visible signal provided by our probe would allow surgeons a direct three-dimensional view in real time, unlike other imaging technologies such as positron emission tomography, computed tomography and magnetic resonance imaging. This is likely to result in superior performance both in detection and surgical removal of metastatic lesions, leading to improvement of cytoreduction efficacy.

In addition, chemical substitution of the β-galactoside moiety with other glycosides has the potential to flexibly target our fluorescence probe to other glycosidases that are enhanced in various diseases, such as β-hexosaminidase in gliomas^32^ and lung cancer^33^, α-mannosidase in breast and colon cancers^31^, β-*N*-acetylgalactosaminidase in colon cancer^34^, and α-fucosidase and β-*N*-acetyl-glucosaminidase in thyroid and gastric cancers^35^. Thus, we believe this simple molecular design strategy is suitable for the development of a series of highly sensitive probes for a range of target enzymes, thereby providing sensitive diagnostics for the corresponding diseases. Further work along this line is in progress, aiming at realizing tailor-made diagnostic guidance.

## Methods

### Synthesis

For synthetic protocols of β-galactosidase fluorescence probes, see the Supplementary Information. Preparative HPLC was performed on HPLC system composed of a pump (PU-2080, JASCO) and detector (MD-2015, JASCO), with an Inertsil ODS-4 (10.0 mm × 250 mm) column (GL Sciences, Inc.). NMR spectra were recorded on a JNM-LA300 instrument (JEOL) at 300 MHz for ^1^H NMR and 75 MHz for ^13^C NMR. Mass spectra were measured with a JMS-T100LC AccuToF (JEOL).

### Optical properties of probes

Ultraviolet–visible spectra were obtained on a V-550 spectrometer (JASCO) and fluorescence spectra were obtained on a FP-6500 fluorescence spectrometer (JASCO). Probes were dissolved in dimethyl sulfoxide (fluorometric grade, Dojindo) to obtain stock solutions. Optical properties of probes were examined in 200 mM sodium phosphate buffer containing 0.03% (v/v) dimethyl sulfoxide as a co-solvent. For determination of fluorescence quantum efficiency (*Φ*_fl_), fluorescein in 0.1 M aqueous NaOH (*Φ*_fl_=0.85) was used as a standard^36^.

### Cell lines and culture

SHIN3 was provided by S. Imai, Nara, Japan. SKOV3 was obtained from American Type Culture Collection. OVCAR3 and OVK18 were obtained from RIKEN Cell Bank. OVCAR4, OVCAR5 and OVCAR8 were provided by the Developmental Therapeutics Program, National Cancer Institute-Frederick, NIH. All cell lines, were grown in RPMI 1640 containing 10% fetal bovine serum (FBS), 100 U ml^−1^ penicillin and 100 μg ml^−1^ streptomycin (all reagents were purchased from Life Technologies). As for preparation of tumor models, OVK18 was grown in MEM (Life Technologies) containing 15% FBS, 100 U ml^−1^ penicillin and 100 μg ml^−1^ streptomycin. All cell lines were maintained at 37 °C in 5% CO_2_.

### Lysate preparation

Cells were cultivated on 100-mm culture dishes at 37 °C in 5% CO_2_ in air. At ~80%–90% confluence, cells were washed twice with 2 ml of DPBS (Life Technologies), and 1 ml of CelLyticM (Sigma) was added. Incubation was continued for 15 min at room temperature on a shaker (180 r.p.m.). The lysed cells were collected and centrifuged for 15 min at 16,000*g* to pellet cellular debris. The supernatant was aliquoted into chilled test tubes and stored at −80 °C. Protein concentration in cell lysates was measured with BCA protein assay kit (Pierce).

### Measurement of β-galactosidase activity

Experiments were performed on 96-well plates (BD Biosciences, 353219). To each well were added 10 μl of cell lysate and 90 μl of 200 mM sodium phosphate buffer (pH 5.0) containing 111 μM TG-βGal (final concentration 100 μM). The plate was incubated at 37 °C for 30 min. To each well, 20 μl of 1.0 M aqueous NaOH was added to quench the reaction. Fluorescence intensity (Ex/Em=492 nm/509 nm) was measured with a microplate reader (SH-8000; Corona, Electric Co., Ltd) and the background intensity of the cell lysate-free control wells was subtracted.

### Confocal imaging

Cells (4 × 10^4^) were plated on eight-chamber plates (Ibidi) and incubated with RPMI 1640 containing 10% FBS for a day. The medium was replaced with phenol red- and serum-free RPMI 1640 containing 10 μM HMRef-βGal. The cells were incubated for 1 h, and differential interference contrast (DIC) and fluorescence images were obtained using a Leica Application Suite Advanced Fluorescence with a TCS SP5 X and a dry objective (× 10, numerical aperture 0.40; Leica). The light source was a white-light laser.

### Measurement of cellular fluorescence intensity

Ovarian cancer cells were plated in 96-well plates at the density of 10,000–20,000 cells per well and cultured at 37 °C in 5% CO_2_ in air for 1–2 days. When cells had reached ~80%–90% confluence, they were rinsed with PBS (pH 7.4; Life Technologies) and to each well was added 100 μl phenol red- and serum-free RPMI 1640 containing 10 μM HMRef-βGal, together with indicated concentrations of β-GA or vehicle alone. After incubation at 37 °C in 5% CO_2_ for 4 h, the fluorescence intensity of each well was measured (Ex/Em=498 nm/518 nm). The background signal at 0 h incubation was subtracted from the observed fluorescence intensity.

### Mouse studies

Female BALB/cAJcl-nu/nu or BALB/cA mice (6–7 weeks old) were purchased from CLEA Japan. All experimental protocols were performed in accordance with the policies of the Animal Ethics Committee of the University of Tokyo.

### Tumour models of peritoneal metastases

The procedure for preparation of tumour mouse models was as previously described^37^,^38^. Briefly, 1 × 10^6^ cells suspended in 300 μl of PBS (pH 7.4) were intraperitoneally injected into female nude mice (BALB/cAJcl-nu/nu, 7–8 weeks old; day 0). In the case of the OVCAR4 models, 5 × 10^6^ cells were injected. Each cell line required a different duration to produce multiple disseminated tumours. Experiments with tumour-bearing mice were performed around day 10 for the SHIN3 model, day 14 for the SKOV3 model, day 21 for the OVCAR4, OVCAR5 and OVCAR8 models, and day 28 for the OVCAR3 and OVK18 models.

### *In vivo* spectral imaging

Mouse models of peritoneal metastasis were injected intraperitoneally with 300 μl of 100 μM probe in PBS (pH 7.4). After 5 min or 1 h, mice were killed by exposure to CO_2_ and the abdominal cavities were exposed. Fluorescence images were obtained with the Maestro *In-Vivo* imaging system (CRi, Inc.). The blue–green filter setting (excitation: 470–510 nm; emission: 530 nm long pass) was used. The tunable filter was automatically stepped in 10-nm increments from 500 to 720 nm, while the camera sequentially captured images at each wavelength interval. ‘Real colour images’ were constructed by combining sequentially captured images with the corresponding colour. The spectral fluorescence images consisting of autofluorescence and probe spectra obtained with Maestro software were left unmixed for visual assessment.

### Dual-colour imaging of metastases

Thirty micrograms of Av-3ROX^30^ was diluted in 300 μl of PBS (pH 7.4) and injected into SHIN3 mouse models. After 3 h, 300 μl of 100 μM HMRef-βGal in PBS (pH 7.4) was intraperitoneally administered to Av-3ROX-treated mice. After 1 h, mice were killed by exposure to CO_2_ and the abdominal cavity was exposed. Fluorescence images were obtained with the Maestro *In-Vivo* imaging system. The blue–green filter setting (excitation: 470–510 nm; emission: 530 nm long pass) and the green filter setting (excitation: 503–555 nm; emission: 580 nm long pass) were used. The tunable filter was automatically stepped in 10-nm increments from 500 to 800 nm. The spectral fluorescence images of autofluorescence and probe spectra obtained with Maestro software were left unmixed for visual assessment.

### Fluorescence endoscopy

A clinical endoscopic system (Evis Exera-II CLV-180, Olympus Corp.) equipped with an in-house-developed fluorescence detection system was used as described previously^18^. The SHIN3 mouse model of peritoneal dissemination was subjected to intraperitoneal administration of 300 μl of 100 μM HMRef-βGal in PBS (pH 7.4). After 1 h, the mice were anaesthetized via inhalation of isoflurane (Abbott Lab.). The endoscope was inserted into the abdominal cavity through a small abdominal incision and the abdominal cavity was inflated with air. Disseminated nodules were monitored in real time in fluorescence or white-light mode. Ex/Em=465–500 nm/516–556 nm.

### Statistical analyses

Statistical comparisons between two samples were made using the unpaired Welch’s *t*-test.

## Author contributions

D.A., M.K., T.N., P.L.K., H.K. and Y.U. designed the research. D.A., M.S., M.K., K.Y., J.H., M.O. and N.K. performed the experiments and analysed the data. T.N., P.L.K., H.K. and Y.U. supervised the project. D.A., M.K. and Y.U. co-wrote the manuscript.

## Additional information

**How to cite this article:** Asanuma, D. *et al*. Sensitive β-galactosidase-targeting fluorescence probe for visualizing small peritoneal metastatic tumours *in vivo*. *Nat. Commun.* 6:6463 doi: 10.1038/ncomms7463 (2015).

## Supplementary Material

Supplementary Information Supplementary Figures 1-7, Supplementary Tables 1-2 and Supplementary Methods

Supplementary Movie 1 Fluorescence endoscopy

Supplementary Movie 2 Fluorescence-guided tumor resection

## Figures and Tables

**Table 1:** Properties of **β**-galactosidase fluorescence probes.