# Supplementary material for: Sensitive β-galactosidase-targeting fluorescence probe for visualizing small peritoneal metastatic tumours in vivo
Source: Nat Commun. 2015 Mar 13;6:6463. doi: 10.1038/ncomms7463 (PMC4382686; doi:10.1038/ncomms7463)
Supplement: Supplementary Information — Supplementary Figures 1-7, Supplementary Tables 1-2 and Supplementary Methods [file ncomms7463-s1.pdf]

Supplementary Figures

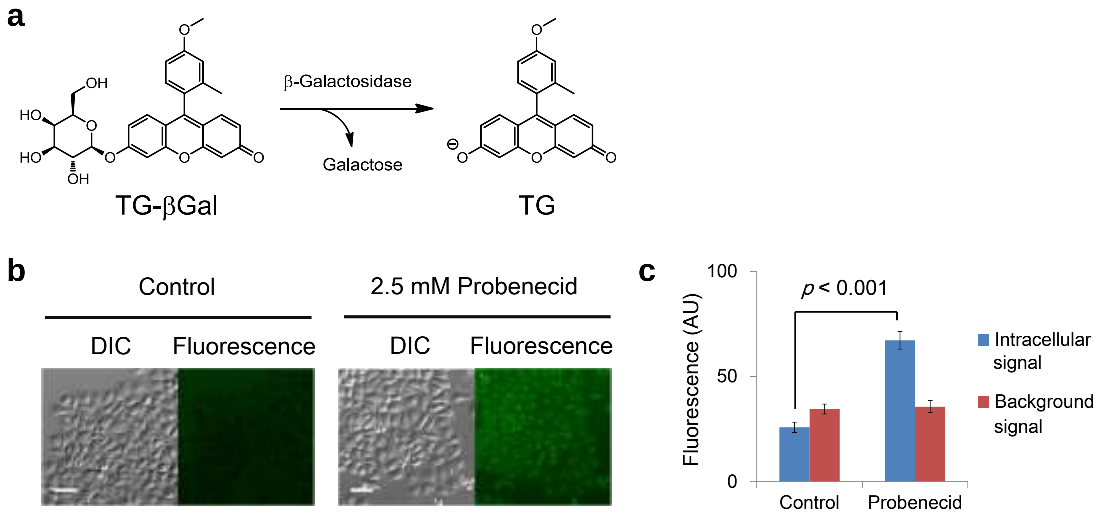

Supplementary Figure 1 | TG-βGal did not visualize β-galactosidase activity in SHIN3 cells due to OATP-mediated efflux activity. (**a**) Enzymatic reaction scheme for TG-βGal with β-galactosidase. (**b**) Confocal imaging of SHIN3 cells with TG-βGal. SHIN3 cells were incubated with 10 μM TG-βGal in the presence or absence of 2.5 mM probenecid for 1 h, and DIC and fluorescence images were obtained. Ex/Em = 490 nm/500-600 nm. Scale bars, 50 μm. (**c**) Fluorescence intensity inside the cells or in the background in the presence or absence of probenecid. Data represent means ± s.d. (n = 10).

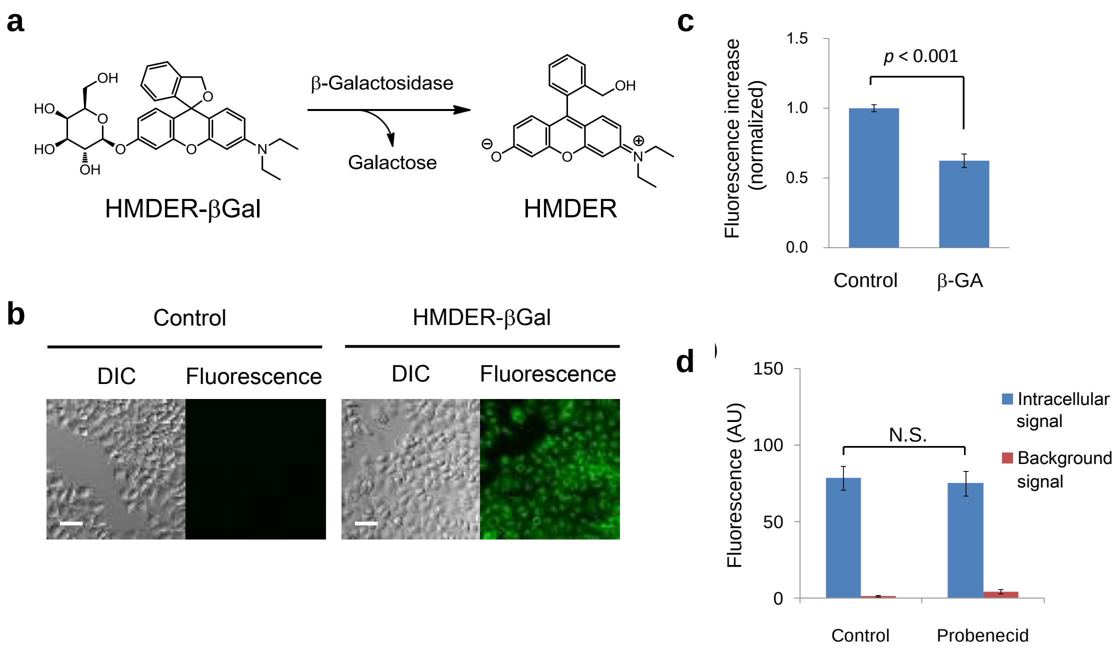

Supplementary Figure 2 | HMDER-βGal detected β-galactosidase activity in SHIN3 cells. (**a**) Enzymatic reaction scheme for HMDER-βGal with β-galactosidase. (**b**) Confocal imaging of SHIN3 cells with or without HMDER-βGal. Cells were incubated with 10 μM HMDER-βGal for 1 h, and DIC and fluorescence images were obtained. Ex/Em = 525 nm/535-600 nm. Scale bars, 50 μm. (**c**) Fluorescence intensity of SHIN3 cells treated with HMDER-βGal in the presence or absence of β-galactosidase inhibitor. Fluorescence intensity of cells on a 96-well plate was measured with a plate reader (Ex/Em = 525 nm/543 nm). Concentrations were 10 μM for HMDER-βGal and 100 μM for β-GA, a β-galactosidase inhibitor. Data represent means ± s.d. (n = 3). (**d**) Fluorescence intensity inside the cells or in the background in the presence or absence of probenecid. Data represent means ± s.d. (n = 10). N.S.: not significant.

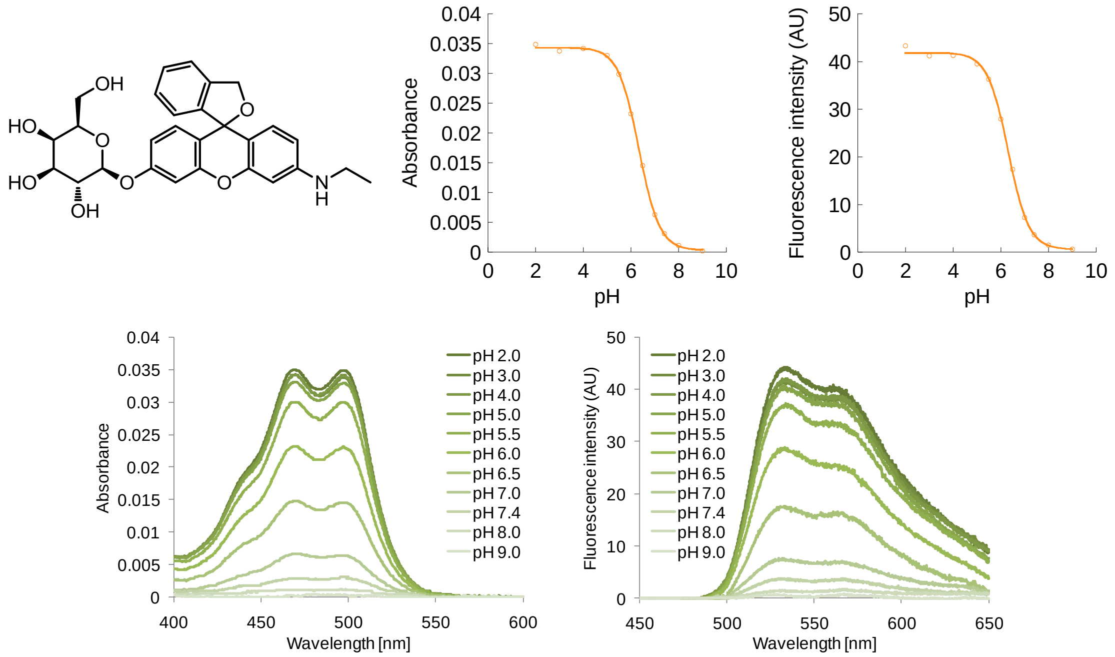

Supplementary Figure 3-1 | Chemical structure, pH profiles and absorption and fluorescence emission spectra of HMRet-βGal. Absorption and fluorescence emission spectra were measured in 200 mM sodium phosphate buffer. Excitation wavelength was 497 nm.

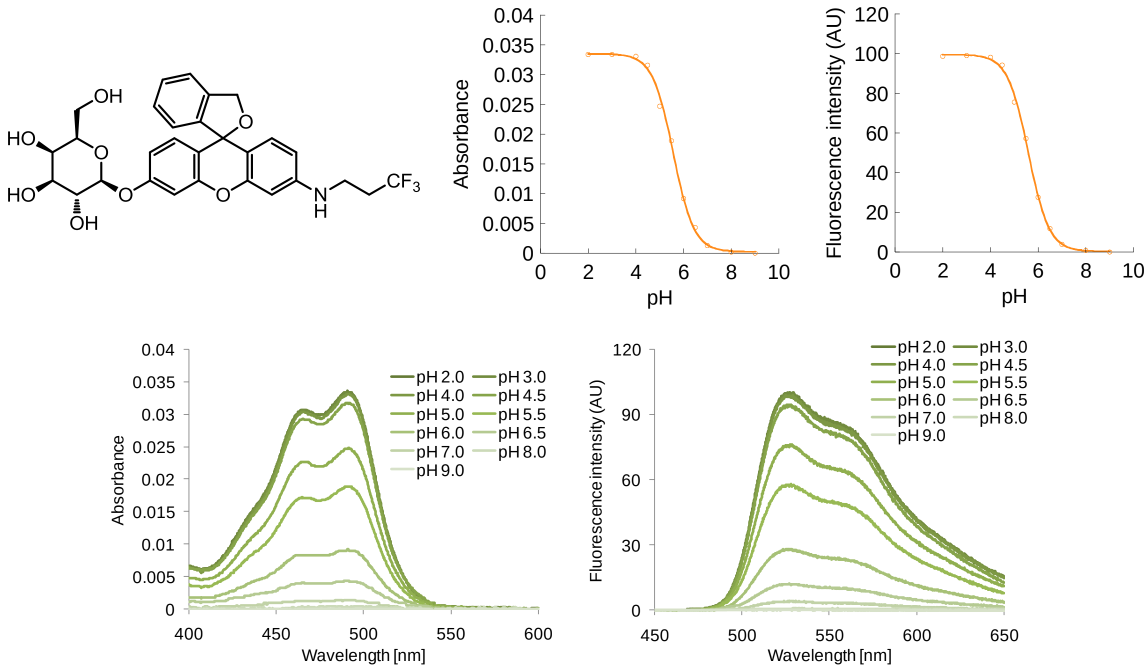

Supplementary Figure 3-2 | Chemical structure, pH profiles and absorption and fluorescence emission spectra of HMRpf-βGal. Absorption and fluorescence emission spectra were measured in 200 mM sodium phosphate buffer. Excitation wavelength was 491 nm.

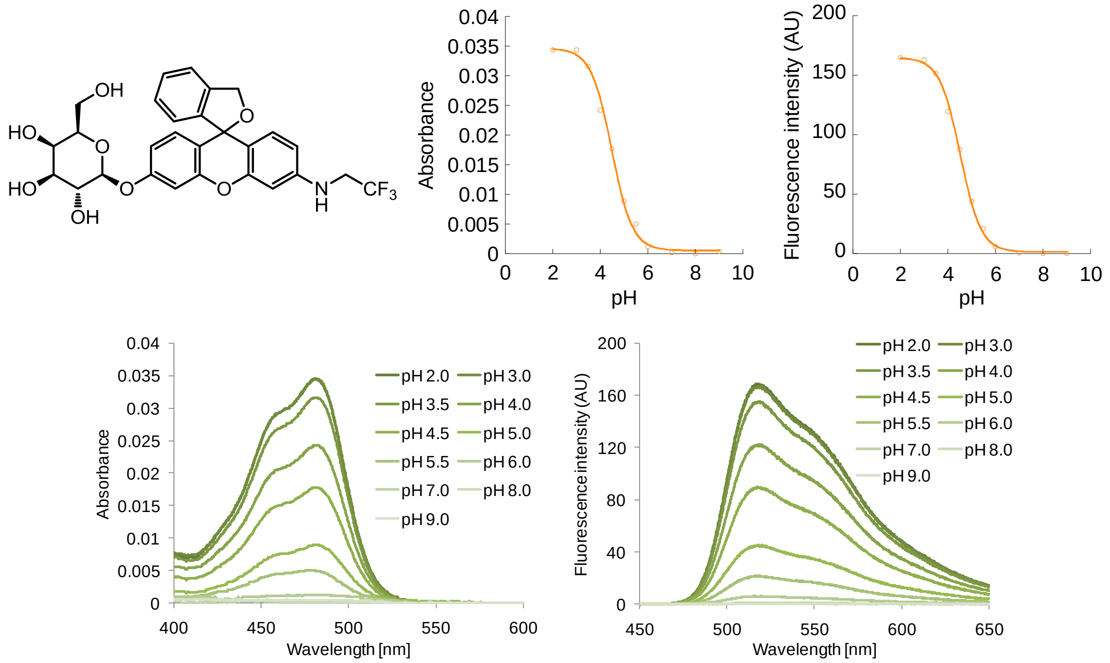

Supplementary Figure 3-3 | Chemical structure, pH profiles and absorption and fluorescence emission spectra of HMRef-βGal. Absorption and fluorescence emission spectra were measured in 200 mM sodium phosphate buffer. Excitation wavelength was 480 nm.

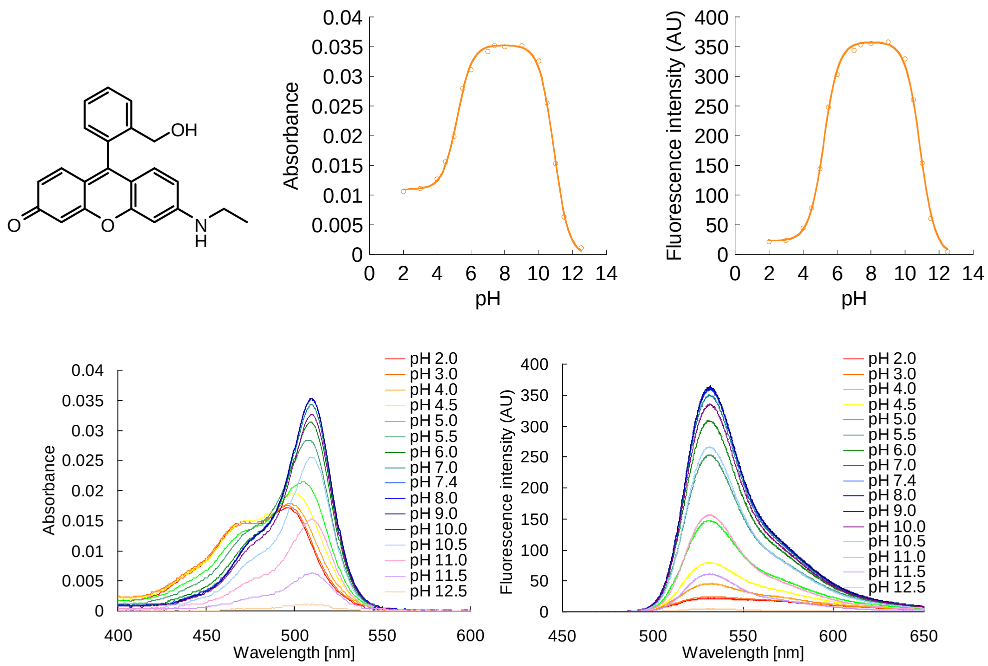

Supplementary Figure 3-4 | Chemical structure, pH profiles and absorption and fluorescence emission spectra of HMRet. Absorption and fluorescence emission spectra were measured in 200 mM sodium phosphate buffer. Excitation wavelength was 510 nm.

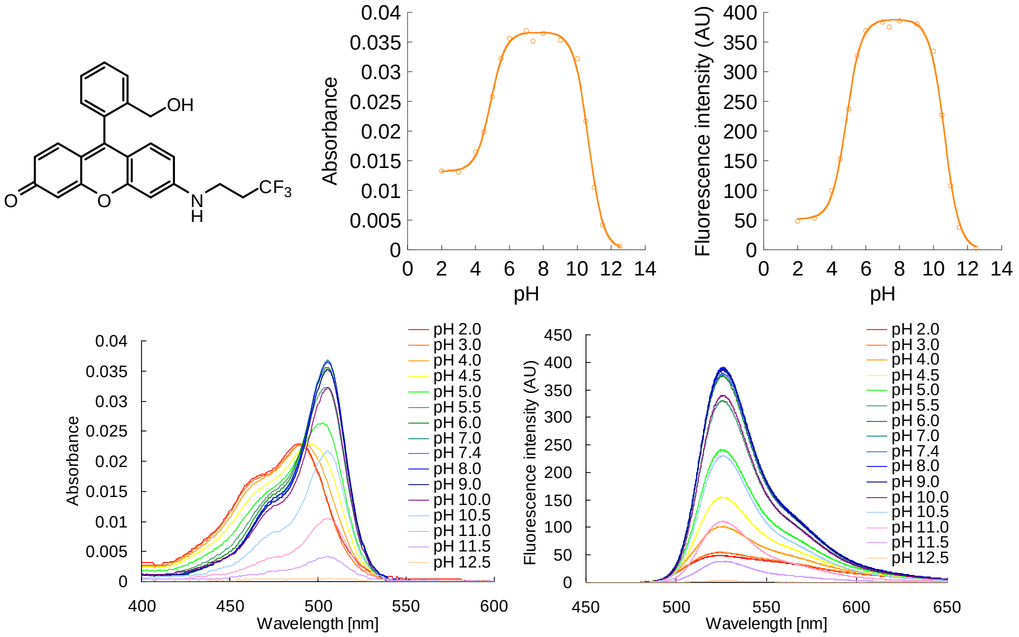

Supplementary Figure 3-5 | Chemical structure, pH profiles and absorption and fluorescence emission spectra of HMRpf. Absorption and fluorescence emission spectra were measured in 200 mM sodium phosphate buffer. Excitation wavelength was 505 nm.

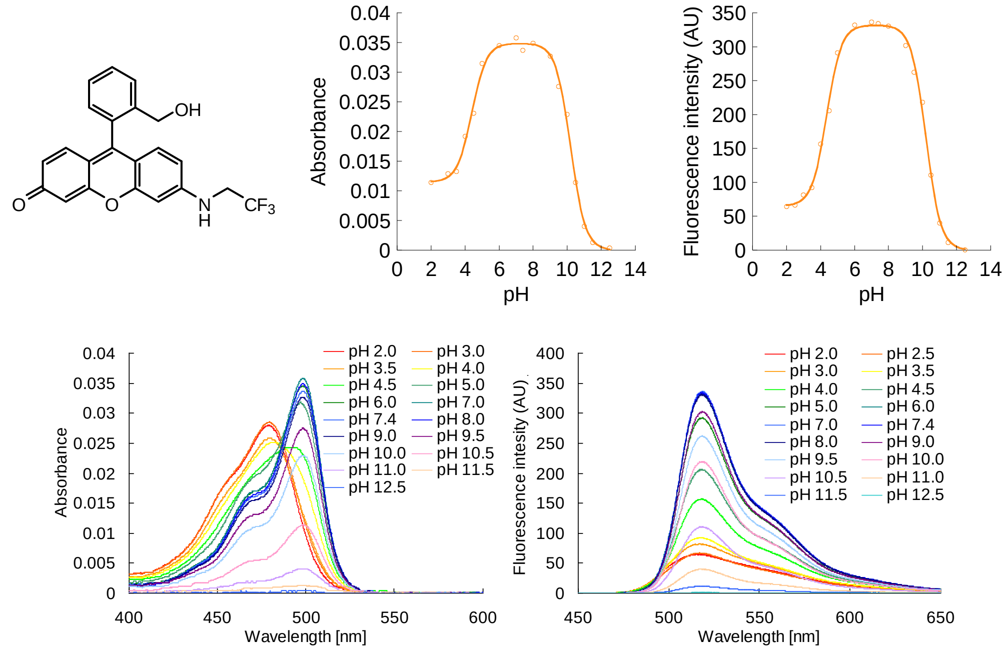

Supplementary Figure 3-6 | Chemical structure, pH profiles and absorption and fluorescence emission spectra of HMRef. Absorption and fluorescence emission spectra were measured in 200 mM sodium phosphate buffer. Excitation wavelength was 498 nm.

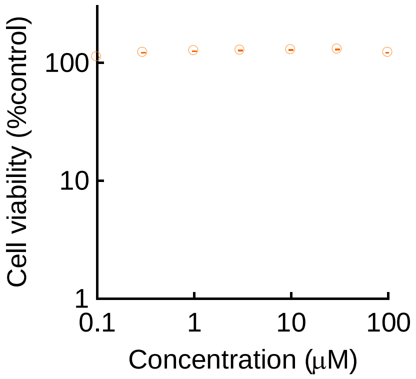

Supplementary Figure 4 | Cell viability test. Survival of SHIN3 cells exposed to the indicated concentrations of HMRef-βGal for 1 h, as determined by MTT assay. Data represent mean + s.d. from a single experiment in triplicate. For details of the protocol, see Supplementary methods.

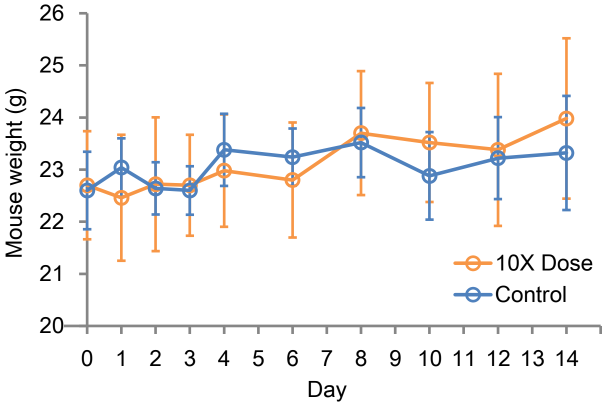

Supplementary Figure 5 | Body weight change of HMRef-βGal-treated mice. At Day 0, female BALB/c mice received intraperitoneal injection of 7 mg/kg HMRef-βGal in PBS (pH 7.4), which is a 10X higher dose than that used for imaging applications. In the control group, vehicle was injected. Data represent mean ± s.d. (n = 5 for each group).

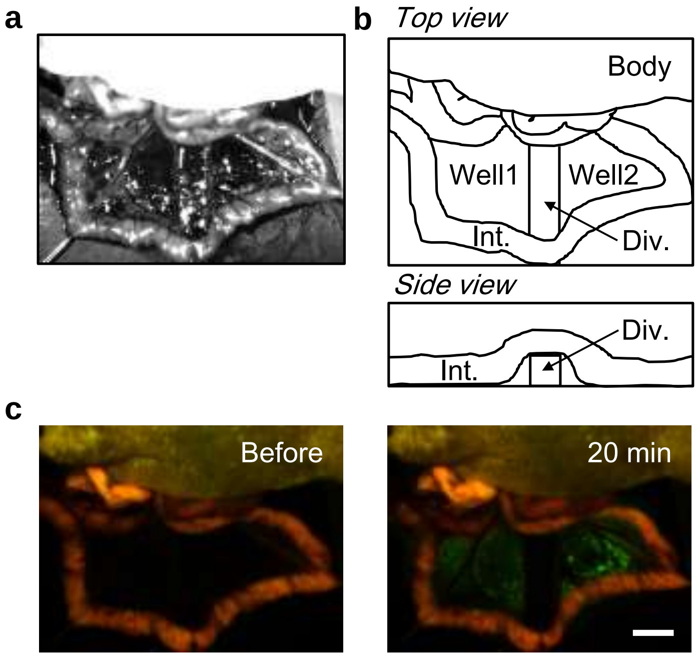

Supplementary Figure 6 | Vital imaging for β-galactosidase inhibition study. (**a**) White light image of the expanded mesentery from an anesthetized, SHIN3-disseminated mouse model. (**b**) Schematic illustration of **a**. Two wells were formed on the mesentery by using a rubber divider (Div.). Int, intestine. (**c**) Real-color fluorescence images of a mouse model before and 20 min after probe administration. Metastases on Well 2 were treated with 100 μM HMRef-βGal in PBS (pH 7.4), whereas those on Well 1 were additionally treated with 10 mM β-GA. Quantitative data are shown in Fig. 3d in the main text. Scale bar, 5 mm.

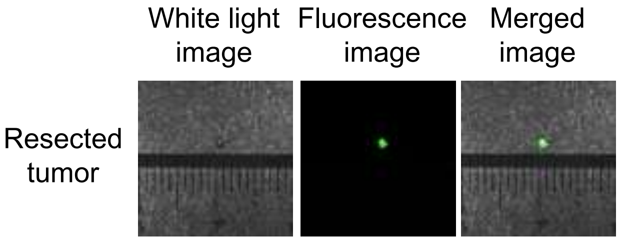

Supplementary Figure 7 | Resected tumor after fluorescence-guided surgery. A mouse model of peritoneal metastasis with SHIN3 cells was intraperitoneally treated with HMRef-βGal in PBS (pH 7.4). After 1 h, the mouse was killed with CO_2 and fluorescence-guided laparotomy for tumor resection was performed. The metastases were clearly visualized, and a 1 mm-sized metastasis was readily recognized and removed from the peritoneal cavity. A fluorescence image of the resected tumor is shown. Ex/Em = 470-510 nm/560 nm. See also Supplementary video S2.

Supplementary Tables

Supplementary Table 1 | Photochemical properties of HMR derivatives

Compound	$\lambda_{abs, max}$ [nm]		$\lambda_{em, max}$ [nm]		ϕ_{fl}	
	pH 2.0	pH 7.4	pH 2.0	pH 7.4	pH 2.0	pH 7.4
HMRet	496	510	531	534	0.283	0.824
HMRet-βGal	497	n.d.	530	n.d.	0.167	n.d.
HMRpf	489	505	524	528	0.559	0.879
HMRpf-βGal	491	n.d.	523	n.d.	0.411	n.d.
HMRef	479	498	515	518	0.716	0.777
HMRef-βGal	480	n.d.	514	n.d.	0.613	n.d.

n.d.: not determined.

Supplementary Table 2 | pK_a and pK_{cycl} values of HMR derivatives

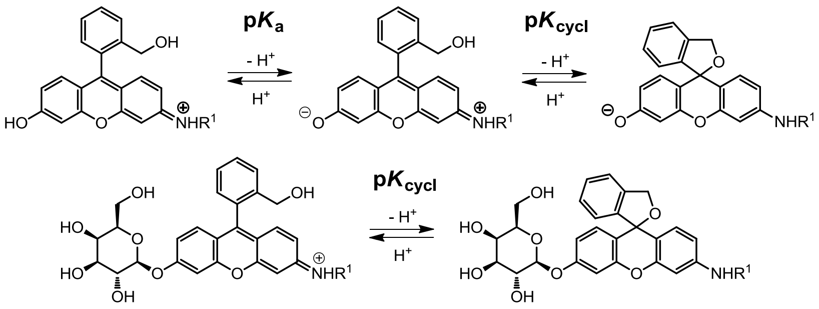

Compound	pK_a	pK_{cycl}
HMRet	5.2	10.9
HMRet-βGal	n.d.	6.3
HMRpf	4.9	10.6
HMRpf-βGal	n.d.	5.6
HMRef	4.4	10.2
HMRef-βGal	n.d.	4.5

n.d.: not determined.

Supplementary Methods

Determination of pK_{cycl} or pK_a values of compounds. Absorption and fluorescence emission spectra of compounds were measured in 200 mM sodium phosphate buffer at different pH values. For compounds with *n* acid-base equilibria (*n* = 1 or 2), pH profiles of absorbance (Abs) or fluorescence intensity (FI) were fitted to the following formula to determine pK_a values.

$$\text{Abs or FI} = \frac{c_0 + \sum_{k=1}^{n} c_k \cdot 10^{k \cdot \mathrm{pH} - \sum_{l=1}^{k} \mathrm{p}K_{al}}}{1 + \sum_{k=1}^{n} 10^{k \cdot \mathrm{pH} - \sum_{l=1}^{k} \mathrm{p}K_{al}}} \qquad \text{(Supplementary Equation 1)}$$

(pK_{a1} < pK_{a2} < ••• < pK_{an}; c_n = constant).

MTT assay. SHIN3 cells were plated in flat-bottomed 96-well plates at the density of 5000 cells/well with 200 μL/well RPMI1640 containing 10% FBS (culture medium). Following incubation (37°C, 5% CO_2 in air) for 1 day, the medium was replaced with 200 μL/well culture medium containing the indicated concentrations of HMR derivatives (1% DMSO as a cosolvent). The cells were then incubated for 1 h. The medium was replaced with 200 μL/well culture medium containing 50 μg/mL MTT, and incubation was continued for 4 h. The medium was replaced with DMSO (100 μL/well), and the absorbance at 570 nm was measured with a microplate reader (SH-9000; Corona, Electric Co., Ltd.).

***In vivo* toxicity test.** Ten female BALB/c mice (8 weeks old) were assigned to two groups of five mice. One group received intraperitoneal injection of 7 mg/kg HMRef-βGal in PBS (pH 7.4), which is a ten times higher dose than that used for imaging applications, while the other group received vehicle alone. Body weight of each mouse was monitored for two weeks.

Vital imaging for β-galactosidase inhibition study. SHIN3 mouse models were anesthetized by intraperitoneal injection of a ketamine-xylazine solution (dose: 80 and 8 mg/kg, respectively). At 10 min post-injection, mice underwent a midline laparotomy and the intestines were carefully pulled out onto a black rubber mat (3 mm thickness) on a plate heater (37 ^{o}C). Using a rubber divider (3 mm × 3 mm × 40 mm), two wells were formed on the mesentery with the aid of forceps (see Supplementary Fig. 6). To one well was added 100 μM HMRef-βGal in PBS (pH 7.4), whereas to the other were added 100 μM HMRef-βGal and 10 mM β-GA. Mice were further anesthetized with isoflurane gas if necessary. Fluorescence spectral images were taken with the Maestro In-Vivo imaging system. The blue-green filter setting (excitation, 470-510 nm; emission, 530 nm long-pass) was used. The tunable filter was automatically stepped in 10-nm increments from 500 to 720 nm, while the camera sequentially captured images at each wavelength interval. Average fluorescence signals at 560 nm on the tumor nodules were evaluated.

Statistical analyses. Statistical comparisons between two samples were made using the unpaired Student's *t* test.

Synthesis of fluorescence probes.

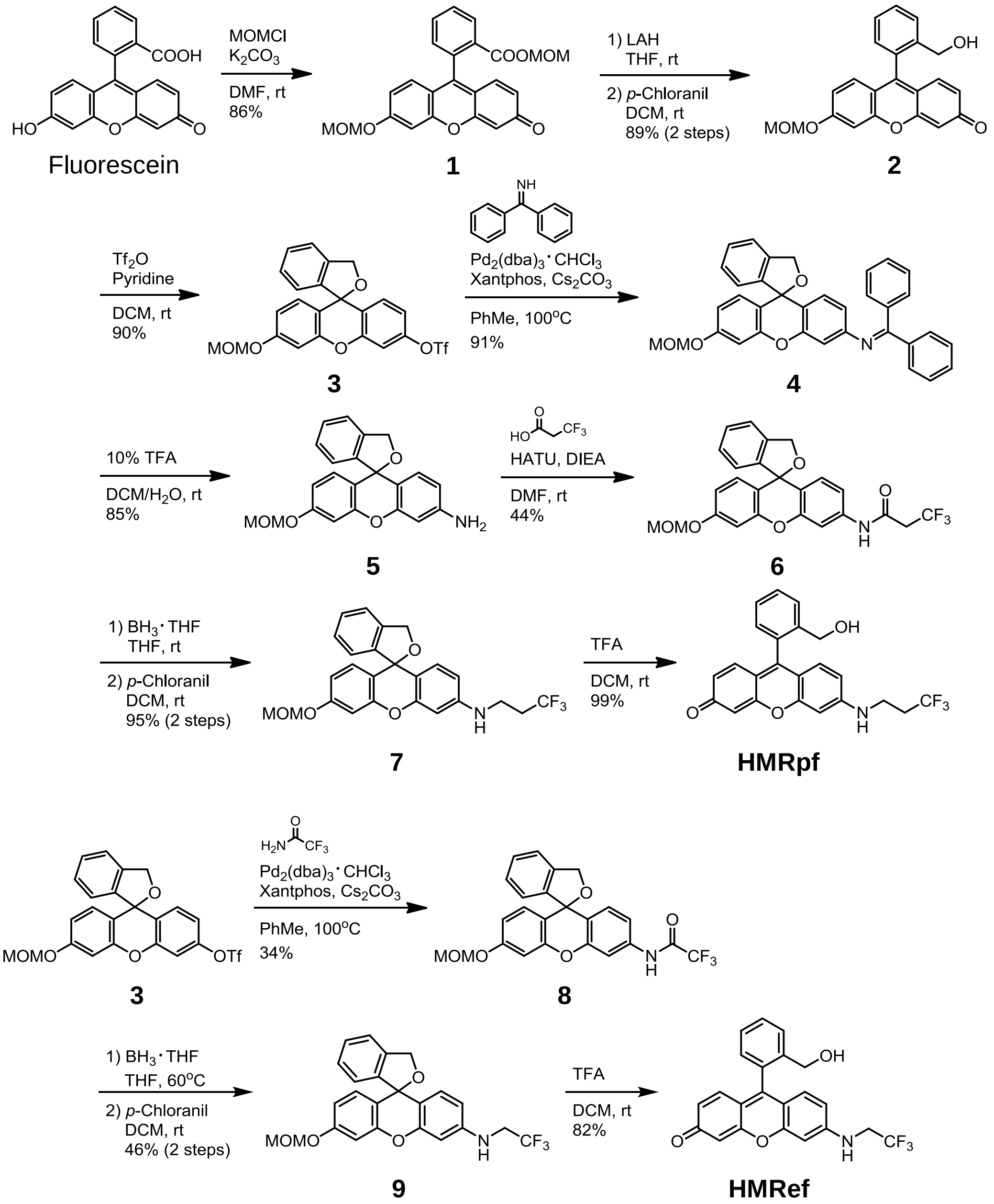

Supplementary Scheme 1 | Syntheses of β-galactosidase fluorescence probes

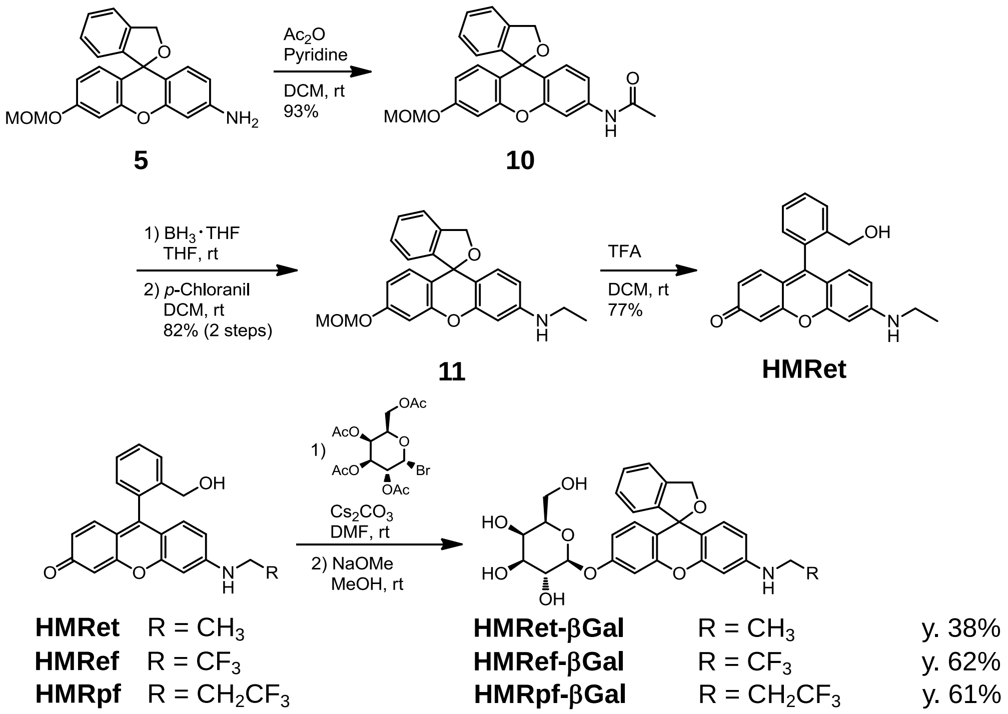

Supplementary Scheme 1 | Syntheses of β-galactosidase fluorescence probes (*continued*)

Methoxymethyl 2-(6-(methoxymethoxy)-3-oxo-3H-xanthen-9-yl)benzoate (1)

Fluorescein (526 mg, 1.58 mmol) was dissolved in 20 mL of DMF. Following addition of K_2CO_3 (547 mg, 3.96 mmol), chloromethyl methyl ether (357 μL, 4.74 mmol) was added dropwise to the reaction solution with intensive stirring. Vigorous stirring was continued for 13 h. To the reaction solution were added 50 mL of ethyl acetate and 10 mL of sat. aq. NH_4Cl. The resulting solution was stirred for 10 min, followed by addition of 40 mL of H_2O. The aqueous layer was acidified to pH ~3 with 85% phosphoric acid, and the organic layer was isolated. The ethyl acetate extracts were washed with 50 mL of brine, dried over anhydrous sodium sulfate, filtered, and concentrated. The crude compound was purified by column chromatography over silica gel using dichloromethane/methanol (95/5) as the eluent, affording **1** (570 mg, 86%) as an orange powder. ^{1}H NMR (300 MHz, $CDCl_3$) δ 3.22 (s, 3H), 3.50 (s, 3H), 5.15-5.21 (m, 2H), 5.27 (s, 2H), 6.45 (d, 1H, J = 1.5 Hz), 6.54 (dd, 1H, J = 9.9, 1.8 Hz), 6.83-6.93 (m, 3H), 7.16 (d, 1H, J = 2.2 Hz), 7.32-7.34 (m, 1H), 7.72-7.76 (m, 1H), 8.30-8.32 (m, 1H). ^{13}C NMR (75 MHz, $CDCl_3$) δ 56.4, 57.7, 91.5, 94.3, 103.1, 105.8, 114.1, 115.5, 117.9, 128.8, 129.7, 130.0 (including two distinct peaks), 130.1, 130.5, 131.3, 133.0, 134.5, 149.7, 153.8, 158.8, 161.4, 164.7, 185.7. HRMS (ESI$^+$) Calcd. for $[M+H]^+$, 421.12873; found, 421.13060 (*Δ* 1.87 mmu).

9-(2-(Hydroxymethyl)phenyl)-6-(methoxymethoxy)-3*H*-xanthen-3-one (2)

1 (390 mg, 0.928 mmol) was dissolved in 50 mL of THF. To the resulting solution was added LAH

(141 mg, 3.72 mmol) in one portion with stirring. The reaction solution was stirred at ambient temperature for 12 h. Methanol (10 mL) was added to quench the reaction, and the solvent was evaporated. The residue was dissolved in 50 mL of dichloromethane and the resulting solution was washed with 50 mL of H_2O containing Rochelle salt. The organic layer was isolated, dried over anhydrous sodium sulfate, filtered, and concentrated. The crude intermediate was dissolved in 20 mL of dichloromethane, and the solution was stirred. Tetrachloro-1,4-benzoquinone (*p*-chloranil) (228 mg, 0.927 mmol) was then added, and stirring was continued for 2 h. After filtration, the reaction solution was washed with H_2O, dried over anhydrous sodium sulfate, filtered, and concentrated. The crude product was purified by column chromatography over silica gel using dichloromethane/methanol (100/0 to wash out impurities, then 95/5) as the eluent, affording **2** (298 mg, 89%) as a yellow powder. ^{1}H NMR (300 MHz, $CDCl_3$) δ 3.46 (s, 3H), 5.16 (d, 2H, *J* = 2.2 Hz), 5.27 (s, 2H), 6.41 (dd, 1H, *J* = 8.8, 2.2 Hz), 6.47 (d, 1H, *J* = 2.2 Hz), 6.69 (dd, 1H, *J* = 8.8, 2.2 Hz), 6.74 (d, 1H, *J* = 8.1 Hz), 6.84 (d, 1H, *J* = 8.8 Hz), 6.86-6.89 (m, 2H), 7.23-7.27 (m, 1H), 7.35-7.37 (m, 2H). ^{13}C NMR (75 MHz, $CDCl_3$) δ 56.0, 71.6, 84.0, 94.2, 102.6, 103.2, 111.9, 112.4, 116.1, 117.8, 120.6, 123.9, 128.1, 128.4, 129.8, 129.9, 138.9, 144.4, 151.4 (including two different peaks), 157.0, 157.7. HRMS (ESI$^+$) Calcd. for $[M+H]^+$, 363.12325; found, 363.12229 (*Δ* -0.95 mmu).

3'-(Methoxymethoxy)-3H-spiro[isobenzofuran-1,9'-xanthen]-6'-yl trifluoromethanesulfonate (3)

2 (150 mg, 0.414 mmol) was dissolved in 10 mL of dichloromethane. After addition of pyridine (134 μL, 1.66 mmol) under stirring, a solution of trifluoromethanesulfonic anhydride (140 μL, 0.829 mmol) in dichloromethane (3 mL) was added to the reaction solution. The resulting mixture was stirred at ambient temperature for 12 h, then concentrated, and the residue was purified by column chromatography over silica gel using dichloromethane as the eluent to give **3** (184 mg, 90%) as a slightly yellow oil. ^{1}H NMR (300 MHz, $CDCl_3$) δ 3.47 (s, 3H), 5.19 (s, 2H), 5.33 (s, 2H), 6.77 (dd, 1H, *J* = 8.8, 2.2 Hz), 6.90-6.94 (m, 4H), 7.07 (d, 1H, *J* = 8.1 Hz), 7.16 (d, 1H, *J* = 2.2 Hz), 7.25-7.32 (m, 1H), 7.36-7.43 (m, 2H). ^{13}C NMR (75 MHz, $CDCl_3$) δ 56.1, 72.5, 82.9, 94.3, 103.3, 109.8, 113.2, 116.2, 117.6, 120.8, 123.7, 125.2, 128.5, 128.6, 129.7, 130.8, 138.7, 144.2, 149.1, 150.7, 151.1, 158.0. HRMS (ESI$^+$) Calcd. for $[M+H]^+$, 495.07253; found, 495.07096 (*Δ* -1.57 mmu).

***N*-(Diphenylmethylene)-3'-(methoxymethoxy)-3H-spiro[isobenzofuran-1,9'-xanthen]-6'-amine (4)**

3 (297 mg, 0.600 mmol), $Pd_2(dba)_3·CHCl_3$ (62.1 mg, 0.0600 mmol; 20 mol% as Pd), xantphos (86.8 mg, 0.150 mmol; 25 mol%) and Cs_2CO_3 (293 mg, 0.900 mmol) were dissolved in 10 mL of toluene under an Ar atmosphere. Benzophenone imine (503 μL, 3.00 mmol) was added, and the resulting mixture was stirred under an Ar atmosphere, first at ambient temperature for 30 min and then at 100

ºC for 23 h. At that time the reaction mixture was allowed to cool to room temperature, diluted with CH_2Cl_2 and filtered through a pad of Celite. The filter cake was washed with CH_2Cl_2. The filtrate was concentrated and the residue was purified by column chromatography over silica gel using AcOEt/n-hexane (1/3) as the eluent to give **4** (289 mg, 91%) as a yellowish oil. ^{1}H NMR (300 MHz, $CDCl_3$) δ 3.43 (s, 3H), 5.14 (s, 2H), 5.27 (s, 2H), 6.41 (dd, 1H, *J* = 8.4, 1.8 Hz), 6.56 (d, 1H, *J* = 2.2 Hz), 6.67 (dd, 1H, *J* = 8.4, 2.6 Hz), 6.76 (d, 1H, *J* = 8.8 Hz), 6.80-6.87 (m, 3H), 7.12-7.48 (m, 11H), 7.72-7.73 (m, 2H). ^{13}C NMR (75 MHz, $CDCl_3$) δ 56.0, 72.1, 83.4, 94.3, 103.2, 108.0, 112.3, 116.7, 118.4, 119.4, 120.5, 123.7, 127.9, 128.0, 128.1, 128.2, 128.8, 129.0, 129.3, 129.6, 130.8, 135.7, 138.7, 139.4, 145.2, 150.6, 151.2, 152.1, 157.6, 168.6. HRMS (ESI^+) Calcd. for $[M+H]^+$, 526.20183; found, 526.20019 (*Δ* -1.64 mmu).

3'-(Methoxymethoxy)-3H-spiro[isobenzofuran-1,9'-xanthen]-6'-amine (5)

4 (176 mg, 0.335 mmol) was dissolved in 10 mL of CH_2Cl_2. To the solution was added 10 mL of H_2O containing 10% (v/v) TFA under stirring. The resulting biphasic mixture was stirred at ambient temperature for 19 h. The reaction solution was basified with 1 M NaOH, and extracted with CH_2Cl_2. The combined CH_2Cl_2 extract was dried over anhydrous sodium sulfate, filtered, and concentrated. The residue was purified by column chromatography over silica gel using AcOEt/n-hexane (1/1) as the eluent to give **5** (103 mg, 85%) as a white solid. ^{1}H NMR (300 MHz, $CDCl_3$) δ 3.46 (s, 3H), 3.74 (br s, 2H), 5.17 (s, 2H), 5.26 (s, 2H), 6.34 (dd, 1H, *J* = 8.8, 2.2 Hz), 6.46 (d, 1H, *J* = 2.9 Hz), 6.68-6.72 (m, 2H), 6.84 (d, 1H, *J* = 8.8 Hz), 6.88-6.91 (m, 2H), 7.24-7.27 (m, 1H), 7.34-7.37 (m, 2H). ^{13}C NMR (75 MHz, $CDCl_3$) δ 56.0, 71.7, 83.6, 94.4, 101.4, 103.3, 111.4, 118.5, 120.5, 123.9, 127.9, 128.2, 129.8, 129.9, 139.3, 144.9, 147.5, 151.4, 151.6, 157.6. HRMS (ESI^+) Calcd. for $[M+H]^+$, 362.13923; found, 362.13621 (*Δ* -3.02 mmu).

3,3,3-Trifluoro-*N*-(3'-(methoxymethoxy)-3H-spiro[isobenzofuran-1,9'-xanthen]-6'-yl)-propanamide (6)

5 (23.5 mg, 0.0650 mmol) and HATU (74.2 mg, 0.195 mmol) were dissolved in 10 mL of DMF. DIEA (68.1 μL, 0.390 mmol) and 3,3,3-trifluoropropionic acid (17.0 μL, 0.195 mmol) were added, and the resulting mixture was stirred at ambient temperature for 24 h, and then concentrated. The residue was purified by column chromatography over silica gel using AcOEt/n-hexane (1/1) as the eluent to give **6** (13.4 mg, 44%) as an off-white solid. ^{1}H NMR (300 MHz, $CDCl_3$) δ 2.97-3.00 (m, 2H), 3.47 (s, 3H), 5.18 (s, 2H), 5.33 (s, 2H), 6.72-6.75 (m, 1H), 6.87-6.89 (m, 4H), 7.10-7.13 (m, 1H), 7.17-7.21 (m, 1H), 7.36-7.40 (m, 2H), 7.85 (s, 1H). ^{13}C NMR (75 MHz, $CDCl_3$) δ 41.6, 42.0, 56.1, 72.0, 83.7, 94.4, 103.4, 107.8, 112.7, 115.8, 117.5, 120.6, 120.7, 123.9, 128.4, 128.6, 129.4, 129.8, 137.9, 138.8, 144.1, 150.6, 151.3, 158.1, 161.0. HRMS (ESI^+) Calcd. for $[M+H]^+$, 494.11913; found, 494.11679 (*Δ* -2.34 mmu).

3'-(Methoxymethoxy)-*N*-(3,3,3-trifluoropropyl)-3H-spiro[isobenzofuran-1,9'-xanthen]-6'-amine (7)

6 (13.8 mg, 0.0293 mmol) was dissolved in 10 mL of THF. BH_3·THF (ca. 1 M in THF, 1 mL) was added under an Ar atmosphere, and the reaction solution was stirred at ambient temperature for 22 h. Methanol was added to quench the reaction, and the reaction solution was concentrated. The residue was dissolved in CH_2Cl_2, and the resulting solution was washed with H_2O, dried over anhydrous sodium sulfate, filtered, and concentrated. The crude intermediate was dissolved in 10 mL of CH_2Cl_2, and the solution was stirred. *p*-Chloranil (7.2 mg, 0.0293 mmol) was then added, and stirring was continued for 9 h. After filtration, the reaction solution was washed with H_2O, dried over anhydrous sodium sulfate, filtered, and concentrated. The crude product was purified by column chromatography over silica gel using dichloromethane/methanol (100/0 to wash out impurities, then > 95/5) as the eluent to give **7** (12.7 mg, 95%) as a yellow solid. ^{1}H NMR (300 MHz, $CDCl_3$) δ 2.40-2.44 (m, 2H), 3.45-3.47 (m, 5H), 5.18 (s, 2H), 5.26 (s, 2H), 6.30 (dd, 1H, *J* = 8.8, 2.2 Hz), 6.39 (d, 1H, *J* = 2.2 Hz), 6.70 (dd, 1H, *J* = 8.8, 2.2 Hz), 6.75 (d, 1H, *J* = 8.8 Hz), 6.84 (d, 1H, *J* = 8.8 Hz), 6.89-6.91 (m, 2H), 7.24-7.28 (m, 1H), 7.35-7.36 (m, 2H). ^{13}C NMR (75 MHz, $CDCl_3$) δ 33.2, 33.6, 37.0, 56.0, 71.8, 83.7, 94.4, 98.5, 103.4, 110.0, 112.3, 114.4, 118.5, 120.6, 124.0, 128.0, 128.3, 129.8, 130.0, 139.4, 144.9, 147.9, 151.5, 151.9, 157.7. HRMS (ESI^+) Calcd. for $[M+H]^+$, 458.15792; found, 458.15863 (*Δ* 0.71 mmu).

9-(2-(Hydroxymethyl)phenyl)-6-((3,3,3-trifluoropropyl)amino)-3H-xanthen-3-one (HMRpf)

7 (4.8 mg, 0.0104 mmol) was dissolved in 5 mL of CH_2Cl_2. TFA (2 mL) was added dropwise, and the reaction mixture was stirred at ambient temperature for 13 h. After addition of CH_2Cl_2, the reaction mixture was washed with 1 M NaOH. The organic layer was isolated, dried over anhydrous sodium sulfate, filtered, and concentrated. The residue was purified by column chromatography over NH silica gel using dichloromethane/methanol (9/1) as the eluent to give **HMRpf** (4.3 mg, 99%) as a red solid. ^{1}H NMR (300 MHz, CD_3OD + NaOD in D_2O) δ 2.42-2.50 (m, 2H), 3.39 (t, 2H, *J* = 7.3 Hz), 5.15 (s, 2H), 6.30-6.32 (m, 3H), 6.37 (d, 1H, *J* = 2.2 Hz), 6.47 (d, 1H, *J* = 8.8 Hz), 6.60 (d, 1H, *J* = 8.8 Hz), 6.83 (d, 1H, *J* = 7.3 Hz), 7.23-7.29 (m, 1H), 7.35-7.37 (m, 2H). ^{13}C NMR (75 MHz, CD_3OD + NaOD in D_2O) δ 34.3, 37.8, 71.7, 87.0, 99.1, 105.4, 110.2, 111.1, 117.3, 121.6, 125.1, 128.8, 129.1, 130.0, 130.9, 140.6, 146.4, 150.3, 153.6, 153.8, 165.3, 174.9. HRMS (ESI^+) Calcd. for $[M+H]^+$, 414.13170; found, 414.13108 (*Δ* -0.62 mmu).

2,2,2-Trifluoro-*N*-(3'-(methoxymethoxy)-3H-spiro[isobenzofuran-1,9'-xanthen]-6'-yl)acetamide (8)

3 (98.9 mg, 0.200 mmol), $Pd_2(dba)_3$·$CHCl_3$ (20.7 mg, 0.0200 mmol; 20 mol% as Pd), xantphos (28.9 mg, 0.050 mmol; 25 mol%), Cs_2CO_3 (326 mg, 1.00 mmol) and 2,2,2-trifluoroacetamide (113 mg,

1.00 mmol) were dissolved in 10 mL of toluene under an Ar atmosphere. The resulting mixture was stirred under an Ar atmosphere, first at ambient temperature for 30 min and then at 100 ºC for 26 h. At that time the reaction mixture was allowed to cool to room temperature, diluted with CH_2Cl_2 and filtered through a pad of Celite. The filter cake was washed with CH_2Cl_2. The filtrate was concentrated and the residue was purified by column chromatography over silica gel using CH_2Cl_2/methanol (95/5) as the eluent to give **8** (31.2 mg, 34%) as a yellowish solid. ^{1}H NMR (300 MHz, $CDCl_3$) δ 3.48 (s, 3H), 5.19 (s, 2H), 5.34 (s, 2H), 6.74 (dd, 1H, *J* = 8.4, 2.6 Hz), 6.86-6.92 (m, 3H), 6.99 (d, 1H, *J* = 8.8 Hz) 7.14 (dd, 1H, *J* = 8.1, 2.2 Hz), 7.24-7.31 (m, 1H), 7.37-7.39 (m, 2H), 7.51 (d, 1H, *J* = 2.2 Hz), 8.10 (br s, 1H). ^{13}C NMR (75 MHz, $CDCl_3$) δ 56.1, 72.3, 83.3, 94.4, 103.3, 108.6, 112.9, 115.7, 117.7, 120.5, 120.8, 122.6, 123.8, 128.4, 128.5, 129.4, 129.8, 129.9, 135.7, 138.8, 144.5, 150.8, 151.0, 158.0. HRMS (ESI^+) Calcd. for $[M+Na]^+$, 480.10348; found, 480.10203 (Δ -1.45 mmu).

3'-(Methoxymethoxy)-*N*-(2,2,2-trifluoroethyl)-3H-spiro[isobenzofuran-1,9'-xanthen]-6'-amine (9)

8 (40.7 mg, 0.0890 mmol) was dissolved in 10 mL of THF. BH_3·THF (ca. 1 M in THF, 5 mL) was added under an Ar atmosphere, and the reaction solution was stirred at 60°C for 40 h. Methanol was added to quench the reaction, and the reaction solution was concentrated. The residue was dissolved in CH_2Cl_2, washed with H_2O, dried over anhydrous sodium sulfate, filtered, and concentrated. The crude intermediate was dissolved in 10 mL of CH_2Cl_2, and the solution was stirred. *p*-Chloranil (21.9 mg, 0.0890 mmol) was added, and stirring was continued for 24 h. After filtration, the reaction solution was washed with H_2O, dried over anhydrous sodium sulfate, filtered, and concentrated. The residue was purified by column chromatography over silica gel using dichloromethane as the eluent to give **9** (18.3 mg, 46%) as a white solid. ^{1}H NMR (300 MHz, $CDCl_3$) δ 3.47 (s, 3H), 3.74-3.80 (m, 2H), 4.06 (t, 1H, *J* = 7.0 Hz), 5.18 (s, 2H), 5.27 (s, 2H), 6.37 (dd, 1H, *J* = 8.8, 2.2 Hz), 6.48 (d, 1H, *J* = 2.9 Hz), 6.70 (dd, 1H, *J* = 8.8, 2.2 Hz), 6.78 (d, 1H, *J* = 8.1 Hz), 6.84 (d, 1H, *J* = 8.8 Hz), 6.89-6.91 (m, 2H), 7.23-7.29 (m, 1H), 7.34-7.39 (m, 2H). ^{13}C NMR (75 MHz, $CDCl_3$) δ 45.9 (q, *J* = 33.8 Hz), 56.1, 71.8, 77.2, 83.6, 94.4, 99.2, 103.4, 109.9, 112.4, 115.4, 118.5, 120.6, 124.0, 128.0, 128.3, 129.8, 130.0, 139.4, 144.8, 147.2, 151.4, 151.8, 157.8. HRMS (ESI^+) Calcd. for $[M+H]^+$, 444.14227; found, 444.14075 (Δ -1.51 mmu).

9-(2-(Hydroxymethyl)phenyl)-6-((2,2,2-trifluoroethyl)amino)-3H-xanthen-3-one (HMRef)

9 (11.4 mg, 0.0257 mmol) was dissolved in 3 mL of CH_2Cl_2, then TFA (2 mL) was added dropwise, and the reaction mixture was stirred at ambient temperature for 19 h. After addition of CH_2Cl_2, the reaction mixture was washed with 1 M NaOH. The organic layer was dried over anhydrous sodium sulfate, filtered, and concentrated. The residue was purified by column chromatography over NH

silica gel using dichloromethane/methanol (95/5) as the eluent to give **HMRef** (8.4 mg, 82%) as a red solid. ^{1}H NMR (300 MHz, CD_3OD + NaOD in D_2O) δ 3.81 (q, 2H, *J* = 9.3 Hz), 6.32 (dd, 1H, *J* = 8.4, 2.6 Hz), 6.35-6.39 (m, 2H), 6.45 (d, 1H, *J* = 2.2 Hz), 6.48 (d, 1H, *J* = 8.1 Hz), 6.61 (d, 1H, *J* = 8.8 Hz), 6.83 (d, 1H, *J* = 7.3 Hz), 7.25-7.27 (m, 1H), 7.35-7.37 (m, 2H). ^{13}C NMR (75 MHz, CD_3OD + NaOD in D_2O) δ 46.1 (q, *J* = 33.6 Hz), 71.7, 86.9, 99.7, 105.3, 110.0, 111.1, 115.6, 117.3, 121.6, 125.1, 128.8, 129.1, 130.0, 130.9, 140.5, 146.4, 149.9, 153.6, 153.7, 170.1. HRMS (ESI^+) Calcd. for $[M+H]^+$, 400.11605; found, 400.11436 (*Δ* -1.70 mmu).

***N*-(3'-(Methoxymethoxy)-3H-spiro[isobenzofuran-1,9'-xanthen]-6'-yl)acetamide (10)**

5 (26.0 mg, 0.0719 mmol) was dissolved in 10 mL of CH_2Cl_2, then pyridine (87.0 μL, 1.08 mmol) was added, and the reaction solution was stirred. Acetic anhydride (34.0 μL, 0.360 mmol) was added dropwise, and the reaction mixture was stirred at ambient temperature for 4 h. After addition of CH_2Cl_2, the reaction solution was washed with H_2O, dried over anhydrous sodium sulfate, filtered, and concentrated. The residue was purified by column chromatography over silica gel using dichloromethane/methanol (95/5) as the eluent to give **10** (26.9 mg, 93%) as a colorless oil. ^{1}H NMR (300 MHz, $CDCl_3$) δ 2.05 (s, 3H), 3.47 (s, 3H), 5.17 (s, 2H), 5.30 (s, 2H), 6.71 (dd, 1H, *J* = 8.8, 2.9 Hz), 6.85-6.89 (m, 4H), 7.05 (dd, 1H, *J* = 8.1, 2.2 Hz), 7.24-7.28 (m, 1H), 7.35-7.37 (m, 3H), 7.73 (br s, 1H). ^{13}C NMR (75 MHz, $CDCl_3$) δ 24.3, 56.0, 72.1, 83.5, 94.4, 103.3, 107.3, 112.5, 115.2, 117.9, 120.1, 120.6, 123.9, 128.2, 128.4, 129.3, 129.8, 138.7, 138.9, 144.6, 150.7, 151.3, 157.8, 168.5. HRMS (ESI^+) Calcd. for $[M+Na]^+$, 426.13174; found, 426.12803 (*Δ* -3.72 mmu).

***N*-Ethyl-3'-(methoxymethoxy)-3H-spiro[isobenzofuran-1,9'-xanthen]-6'-amine (11)**

10 (37.0 mg, 0.0917 mmol) was dissolved in 5 mL of THF. BH_3·THF (ca. 1 M in THF, 1 mL) was added under an Ar atmosphere, and the reaction solution was stirred at ambient temperature for 11 h. Methanol was added to quench the reaction, and the reaction solution was concentrated. The residue was dissolved in CH_2Cl_2, and the resulting solution was washed with H_2O, dried over anhydrous sodium sulfate, filtered, and concentrated. The crude intermediate was dissolved in 10 mL of CH_2Cl_2, and the resulting solution was stirred. *p*-Chloranil (22.5 mg, 0.0915 mmol) was added, and stirring was continued for 4 h. After filtration, the reaction solution was washed with H_2O, dried over anhydrous sodium sulfate, filtered, and concentrated. The crude product was purified by column chromatography over silica gel using AcOEt/n-hexane (1/1) as the eluent to give **11** (32.9 mg, 92%) as a red solid. ^{1}H NMR (300 MHz, CD_3OD) δ 1.22 (t, 3H, *J* = 7.3 Hz), 3.11 (q, 2H, *J* = 7.3 Hz), 5.17 (s, 2H), 5.21 (s, 2H), 6.32-6.35 (m, 2H), 6.63-6.66 (m, 2H), 6.78-6.83 (m, 3H), 7.23-7.26 (m, 1H), 7.35-7.38 (m, 2H). ^{13}C NMR (75 MHz, CD_3OD) δ 14.7, 39.1, 56.3, 72.5, 85.7, 95.5, 98.7, 104.1, 111.1, 113.1, 119.6, 121.5, 121.8, 124.8, 129.1, 129.3, 130.6, 131.0, 140.3, 145.6, 146.1, 151.7, 153.1, 159.3. HRMS (ESI^+) Calcd. for $[M+H]^+$, 390.17053; found, 390.17098 (*Δ* 0.45 mmu).

6-(Ethylamino)-9-(2-(hydroxymethyl)phenyl)-3H-xanthen-3-one (HMRet)

11 (23.4 mg, 0.0601 mmol) was dissolved in 7 mL of CH_2Cl_2. TFA (3 mL) was added dropwise, and the reaction mixture was stirred at ambient temperature for 13 h. After addition of CH_2Cl_2, the reaction mixture was washed with 1 M NaOH. The organic layer was isolated, dried over anhydrous sodium sulfate, filtered, and concentrated. The residue was purified by column chromatography over NH silica gel using dichloromethane/methanol (95/5) as the eluent to give **HMRet** (16.0 mg, 77%) as a red solid. ^{1}H NMR (300 MHz, CD_3OD + NaOD in D_2O) δ 1.23 (t, 3H, *J* = 7.0 Hz), 3.11 (q, 2H, *J* = 7.0 Hz), 5.39 (s, 2H), 6.30-6.33 (m, 3H), 6.38 (d, 1H, *J* = 2.2 Hz), 6.47 (d, 1H, *J* = 8.8 Hz), 6.56 (d, 1H, *J* = 8.8 Hz), 6.82 (d, 1H, *J* = 7.3 Hz), 7.23-7.28 (m, 1H), 7.34-7.37 (m, 2H). ^{13}C NMR (75 MHz, CD_3OD + NaOD in D_2O) δ 14.8, 39.2, 71.6, 87.1, 99.2, 105.3, 110.5, 111.2, 114.3, 117.2, 121.6, 125.1, 128.8, 129.1, 130.1, 130.6, 140.5, 146.4, 151.3, 153.7, 153.8, 170.1. HRMS (ESI$^+$) Calcd. for $[M+Na]^+$, 368.12626; found, 368.12497 (Δ -1.29 mmu).

(2S,3R,4S,5R,6R)-2-((6'-(Ethylamino)-3H-spiro[isobenzofuran-1,9'-xanthen]-3'-yl)oxy)-6-(hydroxymethyl)tetrahydro-2H-pyran-3,4,5-triol (HMRet-βGal)

A solution of **HMRet** (7.3 mg, 0.0211 mmol) and Cs_2CO_3 (543 mg, 1.66 mmol) in 5 mL of DMF was stirred at ambient temperature. A solution of 2,3,4,6-tetra-O-acetyl-alpha-D-galactopyranosyl bromide (206 mg, 0.500 mmol) in DMF (0.5 mL) was added dropwise, and the reaction mixture was stirred at ambient temperature in the dark for 12 h, then filtered through a Celite pad, and evaporated. The residue was extracted with dichloromethane three times, and the combined organic layer was washed with brine, dried over Na_2SO_4, and evaporated. The residue was purified by column chromatography over silica gel using CH_2Cl_2/MeOH (9/1) as the eluent to give a 2,3,4,6-tetra-*O*-acetyl-β-D-galactopyranosylated derivative. This was dissolved in 10 mL of methanol, and 100 μL of 28% NaOMe in MeOH was added. The mixture was stirred at ambient temperature for 5 h, and then the reaction was quenched by adding 1 mL of H_2O. The reaction mixture was concentrated, and column chromatography over silica gel using CH_2Cl_2/MeOH (9/1) as the eluent afforded **HMRet-βGal** (4.1 mg, 38%) as a colorless oil. ^{1}H NMR (300 MHz, CD_3OD) δ 1.23 (t, 3H, *J* = 7.0 Hz), 3.12 (q, 2H, *J* = 7.0 Hz), 3.58 (dd, 1H, *J* = 9.5, 2.9 Hz), 3.68-3.82 (m, 5H), 3.91 (d, 1H, *J* = 2.9 Hz), 5.23 (s, 2H), 6.33-6.37 (m, 2H), 6.64 (d, 1H, *J* = 8.8 Hz), 6.79-6.83 (m, 3H), 6.93 (d, 1H, *J* = 1.5 Hz), 7.25-7.28 (m, 1H), 7.37-7.40 (m, 2H). ^{13}C NMR (75 MHz, CD_3OD) δ 14.7, 39.1, 62.4, 70.2, 72.2, 72.5, 74.9, 77.1, 85.7, 98.7, 102.8, 104.9, 111.2, 113.3, 113.4, 113.5, 121.8, 124.8, 129.2, 129.4, 130.6, 131.0, 140.3, 146.2, 151.8, 152.9, 153.1, 159.7. HRMS (ESI$^+$) Calcd. for $[M+H]^+$, 530.17909; found, 530.17437 (Δ -4.72 mmu).

(2R,3R,4S,5R,6S)-2-(Hydroxymethyl)-6-((6'-((3,3,3-trifluoropropyl)amino)-3H-spiro[isobenzofuran-1,9'-xanthen]-3'-yl)oxy)tetrahydro-2H-pyran-3,4,5-triol (HMRpf-βGal)

This compound was synthesized using **HMRpf** as described above. 61% yield. ^{1}H NMR (300 MHz, CD_3OD) δ 2.44-2.49 (m, 2H), 3.40 (t, 2H, *J* = 7.3 Hz), 3.55-3.61 (m, 2H), 3.72-3.82 (m, 4H), 3.90 (d, 1H, *J* = 2.9 Hz), 5.24 (s, 2H), 6.35-6.37 (m, 2H), 6.67-6.69 (m, 1H), 6.76-6.83 (m, 3H), 6.93-6.94 (m, 1H), 7.25-7.28 (m, 1H), 7.37-7.40 (m, 2H). HRMS (ESI^+) Calcd. for $[M+Na]^+$, 598.16647; found, 598.16498 (*Δ* -1.49 mmu).

(2R,3R,4S,5R,6S)-2-(Hydroxymethyl)-6-((6'-((2,2,2-trifluoroethyl)amino)-3H-spiro[isobenzofuran-1,9'-xanthen]-3'-yl)oxy)tetrahydro-2H-pyran-3,4,5-triol (HMRef-βGal)

This compound was synthesized using **HMRef** as described above. 62% yield. ^{1}H NMR (300 MHz, CD_3OD) δ 3.59 (dd, 1H, *J* = 9.9, 3.3 Hz), 3.70-3.84 (m, 7H), 3.90 (d, 1H, *J* = 3.7 Hz), 5.24 (s, 2H), 6.44 (dd, 1H, *J* = 8.4, 2.6 Hz), 6.50 (d, 1H, *J* = 2.2 Hz), 6.70 (d, 1H, *J* = 8.8 Hz), 6.81-6.83 (m, 3H), 6.93-6.96 (m, 1H), 7.24-7.30 (m, 1H), 7.35-7.45 (m, 2H). ^{13}C NMR (75 MHz, CD_3OD) δ 46.0 (q, *J* = 33.8 Hz), 62.4, 70.2, 72.2, 72.7, 74.8, 77.1, 85.5, 99.4, 102.8, 104.9, 110.8, 113.6, 113.7, 114.9, 120.1, 121.9, 124.8, 129.3, 129.4, 130.9, 131.0, 140.3, 146.1, 150.3, 152.7, 153.0, 159.7. HRMS (ESI^+) Calcd. for $[M+Na]^+$, 584.15082; found, 584.14926 (*Δ* -1.56 mmu).
